# LncRNA-PVT1 was identified as a key regulator for TMZ resistance and STAT-related pathway in glioma

**DOI:** 10.1186/s12885-023-10937-9

**Published:** 2023-05-18

**Authors:** Yusheng Chen, Fengjin Ma, Zhe Zhang, Yang Guo, Hanwei Shen, Hang Chen

**Affiliations:** 1 Department of Neurosurgery, Henan Provincial People’s Hospital, People’s Hospital of Zhengzhou University, People’s Hospital of Henan University, No.7 WeiWu Street, 450003 Zhengzhou, Zhengzhou, China; 2 Department of Intensive Care Unit, The Third People’s Hospital of Zhengzhou, Zhengzhou, China

**Keywords:** Glioma, PVT1, Chemoresistance, JAK/STAT, Survival

## Abstract

**Background:**

PVT1, a previously uncharacterized lncRNA, was identified as a critical regulator involved in multiple functions in tumor, including cell proliferation, cell motility, angiogenesis and so on. However, the clinical significance and underlying mechanism of PVT1 was not be fully explored in glioma.

**Methods:**

In this study, 1210 glioma samples with transcriptome data from three independent databases (CGGA RNA-seq, TCGA RNA-seq and GSE16011 cohorts) were enrolled in this study. Clinical information and genomic profiles containing somatic mutations and DNA copy numbers were collected from TCGA cohort. The R software was performed for statistical calculations and graphics. Furthermore, we validated the function of PVT1 in vitro.

**Results:**

The results indicated that higher PVT1 expression was associated with aggressive progression of glioma. Cases with higher PVT1 expression always accompanied by PTEN and EGFR alteration. In addition, functional analyses and western blot results suggested that PVT1 inhibited the sensitivity of TMZ chemotherapy via JAK/STAT signaling. Meanwhile, knockdown of PVT1 increased the sensitivity of TZM chemotherapy in vitro. Finally, high PVT1 expression was associated with reduced survival time and may serve as a strong prognostic indicator for gliomas.

**Conclusions:**

This study demonstrated that PVT1 expression strongly correlated with tumor progression and chemo-resistance. PVT1 may become a potential biomarker for the diagnosis and treatment in glioma.

**Supplementary Information:**

The online version contains supplementary material available at 10.1186/s12885-023-10937-9.

## Background

Glioma is the most prevalent malignant tumor in central nerve system in adults [1]. The standards of care in glioma are the maximally safe surgical resection followed by radiotherapy and chemotherapy with temozolomide (TMZ)[2, 3]. Regardless of multidisciplinary treatment, patients will experience tumor progression and recurrence with nearly universal mortality [4, 5]. As a disease with such a poor prognosis, the advances on basic and clinical researches of glioma to improve survival and preserve the quality of life are urgently needed.

In recent years, the roles of lncRNAs in oncogenesis were widely studied, as well as their potential in development of new innovative therapies in future [6, 7]. PVT1 encodes for a lncRNA, called lncPVT1 that has been shown to be associated with several tumors [8, 9]. Since then, PVT1 has repeatedly emerged in many profiling studies as a prominently dysregulated lncRNA in tumors.

Previous studies suggested that PVT1 could promote the occurrence and development of cancers by affecting cell proliferation, migration, invasion and apoptosis [10]. Using 97 patients’ data at Yiwu Central Hospital, Fang et al. found that PVT1 was highly expressed in tumor and was an unfavorable prognosis factor for glioma [11]. However, the clinical and genetic features related PVT1 was not be clarified yet. Meanwhile, there have been few reports illustrating the role of PVT1 in TMZ chemoresistance. Thus, deeply investigating the molecular mechanism and functional diversity of PVT1 in glioma may help to get a potential therapeutic target in glioma. In this study, we gathered genomic and transcriptomic profiles from three independent cohorts (TCGA, CGGA and GSE16011) to comprehensively depict the role of PVT1 in gliomas. Interestingly, we found that PVT1 was not only played an important role in tumor progression, but was also tightly related to the sensitivity of TMZ chemotherapy via JAK/STAT signaling in glioma. This study was the first integrative study to characterize PVT1 expression in glioma in clinical and molecular aspects.

## Methods

### Patients and cohorts

In total, the 1210 eligible patients who were diagnosed with glioma according to 2016 World Health Organization classification for the central nervous system tumors were included in this study. The transcriptome data and corresponding clinical information were collected from the Chinese Glioma Genome Atlas (CGGA) cohort (n = 325) (http://www.cgga.org.cn), The Cancer Genome Atlas (TCGA) cohort (n = 627) (http://cancergenome.nih.gov/) and the GSE16011 (n = 258) (https://www.ncbi.nlm.nih.gov/geo/query/acc.cgi? acc = gse16011). The somatic mutation and copy-number alterations (CNAs) were obtained from TCGA cohort. Overall survival (OS) was calculated from the date of diagnosis until death or the end of follow-up. The clinicopathologic characteristics of the patients from three datasets were shown in Table 1.

**Table 1:** The clinical and molecular characteristics of glioma in three independent datasets

	CGGA dataset (n = 325)	TCGA dataset (n = 627)	GSE16011 (n = 258)
Age			
≤40 years	138	242	71
>40 years	187	385	187
Gender			
Male	203	363	180
Female	122	264	78
WHO grade			
Grade II	109	221	23
Grade III	72	245	84
Grade IV	144	161	151
IDH Status			
Mutant	167	390	77
Wild-type	158	237	130
1p/19q Status			
Codel	64	154	48
Intact	261	457	104
Molecular subtype			
Proneural	102	136	91
Neural	81	71	26
Classical	74	83	57
Mesenchymal	68	92	84

### Cell culture and transfection

Human glioma cell lines U87 and LN229 were purchased from the American Type Culture Collection (ATCC, Manassas, VA, USA). All of these cell lines were cultured in DMEM culture medium with added FBS at 10% final concentration and added antibiotics penicillin and streptomycin at 1% final concentrations (Life Technologies, China). Cells were cultured in certified incubators at 37℃ under humidified conditions and 5% CO2. The small interference RNA (siRNA) and negative control (NC) of PVT1 were synthesized by Santa-cruz Biotechnology Co, Inc. In accordance with the manufacturer’s instructions, U87 and LN229 cell lines were transfected with siRNA or NC using the INTERFER in Transfection reagent (Polyplus-transfection Co., Ltd.) when the cell density reached 30–50%.

### Western blot analysis

Total cellular proteins were lysed by RIPA lysis buffer (Beyotime, China). Total protein was extracted and quantified with the BCA Protein Assay kit (Beyotime Institute of Biotechnology) according to the manufacturer’s protocol. Equal quantities of total protein (40 µg) from the cell lysates were subjected to 10% SDS-PAGE to separate the proteins and then transferred to a polyvinylidene fluoride (PVDF) membrane (EMD Millipore, Billerica, MA, USA). After incubation with antibodies specific for JAK3 (1:1000, cat.no.ab45141, Abcam, USA), STAT3 (1:2000,cat.no.10253-2-AP, Proteintech, USA) or GAPDH (1:5000, cat.no.60004-1-Ig, Proteintech, USA), the membranes were then incubated with peroxidase (HRP)-conjugated secondary antibody. After washes, bands were detected using the Chemi-DocTM XRS + (Bio-Rad, USA). GAPDH was used as a loading control.

### Cell scratch assays

U87 and LN229 cells were seeded in 6-well plates (1 × 10^5^ cells per well) and incubated in a 37 °C, 5% CO2 incubator. At 48 h after transfected with PVT1 siRNA or a NC siRNA, the cell monolayer was scraped with a sterile 200-µL pipette tip. Then, fresh medium without serum was added to the plates and each well was photographed to mark the “zero point” of migration. After completion of 24 h incubation, the samples were washed twice very gently with PBS (Gibco; Thermo Fisher Scientific). Each well was photographed using computer-assisted microscopy. Phase-contrast images were taken at the beginning (0 h) and after 24 h under ×100 magnitude microscope.

### Cell migration assays

Cell migration assay was performed using 24-well transwell chambers (8 μm pore size, Corning, Corning, NY, USA). After the transfection with PVT1 siRNA and NC siRNA, approximately 1 × 10^5^ cells were seeded into upper chambers with serum-free medium in triplicate and medium with 20% FBS was added into the lower chamber. After incubation for 5 h, U87 and LN229 cells were fixed in 4% paraformaldehyde solution for 10 min, followed by staining with 5% crystal violet solution. Cell images were captured under an inverted microscope (IX51; Leica Microsystems GmbH, Wetzlar, Germany) in 6 randomly selected fields.

### Real-time PCR (RT-PCR)

Moreover, 15 samples, including 5 WHO grade II gliomas, 5 WHO grade III gliomas and 5 WHO grade IV gliomas, were collected to assess differentially expression of PVT1 by real-time quantitative PCR. Total RNA was isolated from glioma samples using TRIzol reagent (Aidalb Biotechnologies Co., Ltd., Beijing, China) following the manufacturer’s instructions. RNA concentration was evaluated by NanoDrop (NanoDrop Technologies; Thermo Fisher Scientific, USA). The RNA quality was estimated by determining the optical density (OD)260/OD280 ratio; values between 1.8 and 2.1 were considered to meet the experimental requirements. Specifically, 1 ug of total RNA was reversely transcribed into cDNA using a Thermo Scientific RevertAid First Strand cDNA Synthesis Kit. The results of real-time PCR were normalized to the corresponding GAPDH mRNA levels and the analysis were performed in triplicate to remove the outliers. The PCR conditions were as follows: Pre-denaturation at 95°C for 10 min, followed by 40 cycles of denaturation at 95°C for 30 sec, annealing at 60°C for 30 sec and extension at 72°C for 30 sec. Relative gene expression was normalized to the expression of β-actin and was calculated by applying the 2-dCt method. The primers were as follows: PVT1: forward: 5’- GGGGAATAACGCTGGTGGAA-3’, PVT1: reverse: 5’- CCCATGGACATCCAAGCTGT-3’, GAPDH: forward: 5’-AGGGCTGCTTTTAACTCTGGT-3’, GAPDH: reverse: 5’-CCCCACTTGATTTTGGAGGGA-3’.

### Cell counting kit-8 (CCK-8) assay

U87 and LN229 cells successfully transfected with siRNA were cultured in medium containing different concentrations of temozolomide (TMZ) (0, 200, 400 and 800 µM) (Sigma-Aldrich; Merck KGaA, Darmstadt, Germany) for 24 h. Then, cells were seeded in 96-well plates at a density of 2 × 10^3^ cells/ well, and cell viability was assessed using the CCK-8 Assay Kit (Dojindo Molecular Technologies, Japan), as instructed by the manufacturer. Samples were measured at OD 490 nm using the Bio-Rad Microplate Reader Model 680 (Bio-Rad, China).

### Gene ontology enrichment analysis

The functional enrichment analysis for PVT1 correlation genes was performed using DAVID software (http://david.abcc.ncifcrf.gov/). Gene set enrichment analysis (GSEA) was performed to identify gene sets of statistical difference using GSEA R package.

### Statistical analysis

The Student’s t-test, one-way ANOVA, or Chi-squared test was used to assess differences in variables between groups. The Kaplan-Meier analysis was performed to evaluated the overall survival of glioma patients using the “survival” and “survminer” packages. The genomic aberration was performed using the “maftools” package. Other statistical computations and figures drawing were performed with several packages (ggplot2, pheatmap and corrgram). All statistical analyses were conducted using R software. A two-sided p value of < 0.05 was considered statistically significant.

## Result

### High expression of PVT1 was associated with glioma progression

**Fig. 1:**
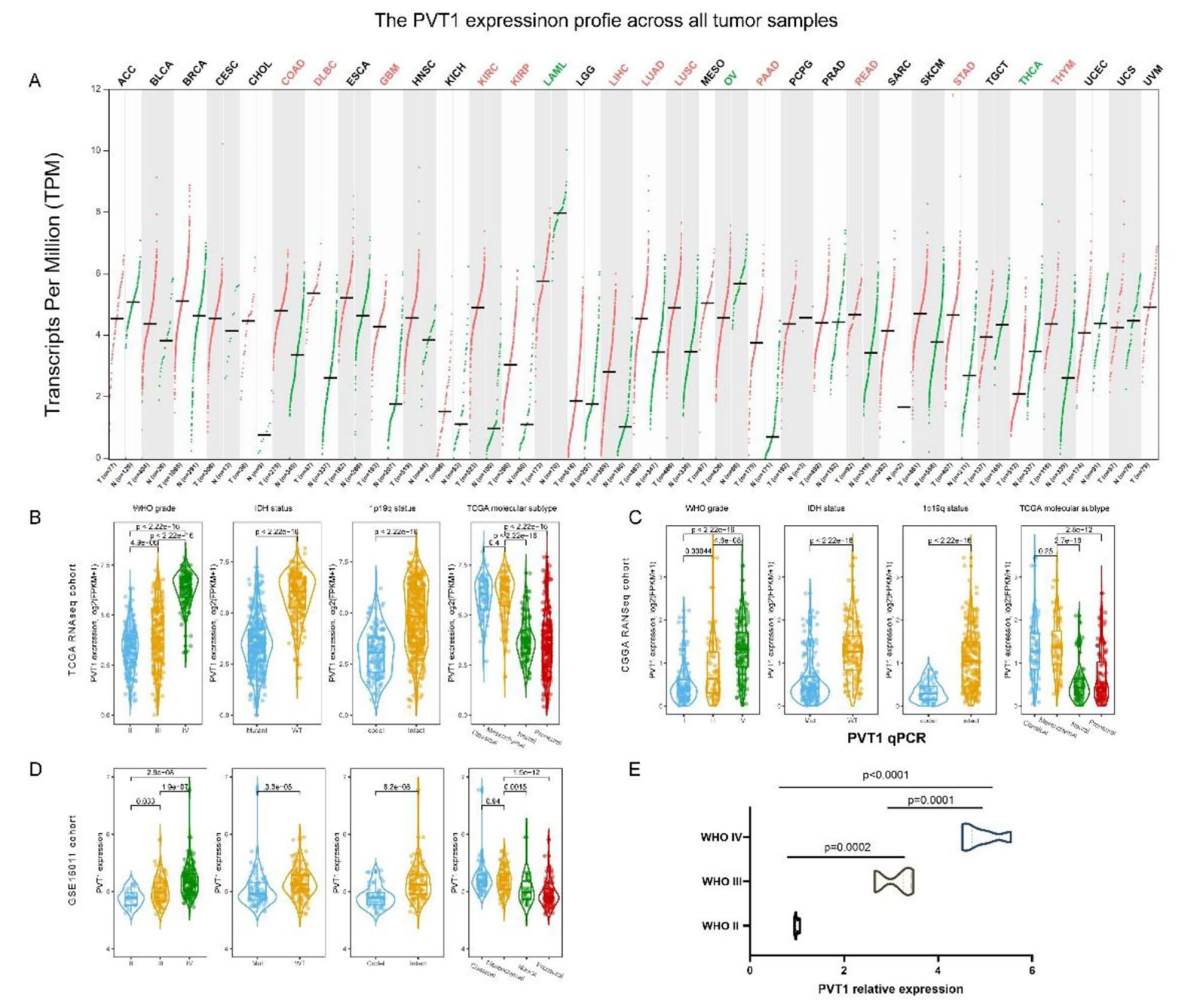
Gene expression pattern of PVT1 in glioma. **(A)** PVT1 expression profiles across all tumor samples in the TCGA cohort (The cancers with red marking indicated that PVT1 expression was significantly upregulated in tumor. The cancers with green marking indicated that PVT1 expression was significantly downregulated in tumor). **(B-D)** Comparison of PVT1 expression level in different WHO grades, IDH status, 1p/19q status and TCGA molecular status in TCGA, CGGA and GSE16011 cohorts. **(E)** qRT-PCR results of PVT1 in different grades of gliomas.

To investigate the role of PVT1 in tumors, we compared the expression pattern of PVT1 between normal tissues and tumor, and found that PVT1 was highly expressed in glioma and many malignancies (Fig. 1A). Then we explored the distribution characteristics of PVT1 expression in glioma, according to WHO grade, IDH status, 1p/19q status and molecular subtype. In TCGA dataset (Fig. 1B), we observed that PVT1 was the highest expressed in the GBM (p < 0.0001) (WHO grade IV) compared with WHO grade II and grade III glioma. Besides, the higher expression of PVT1 was detected in IDH wildtype (p < 0.0001) or 1p/19q (p < 0.0001) intact glioma, which involved in the malignant progression of glioma. Meanwhile, glioma in mesenchymal and classical (p < 0.0001) molecular subtypes had higher PVT1 expression. Similar results were well validated in CGGA (Fig. 1C) and GSE16011 (Fig. 1D) cohorts and indicated higher PVT1 expression level always associated with the higher malignance of glioma. Furthermore, the qPCR experiment (Fig. 1E) of glioma patients (WHO II–IV grade) showed that PVT1 was the highest in WHO IV patients and lowest in WHO II patients (p < 0.0001). Among the identified transcriptome subtypes, the mesenchymal and classical subtype gliomas tended to survive shorter and were associated with chemotherapy resistance [12, 13]. Our results indicated that higher PVT1 expression was closely related with glioma malignant progression and chemotherapy resistance.

### PVT1 associated clinical and molecular features in glioma

**Fig. 2:**
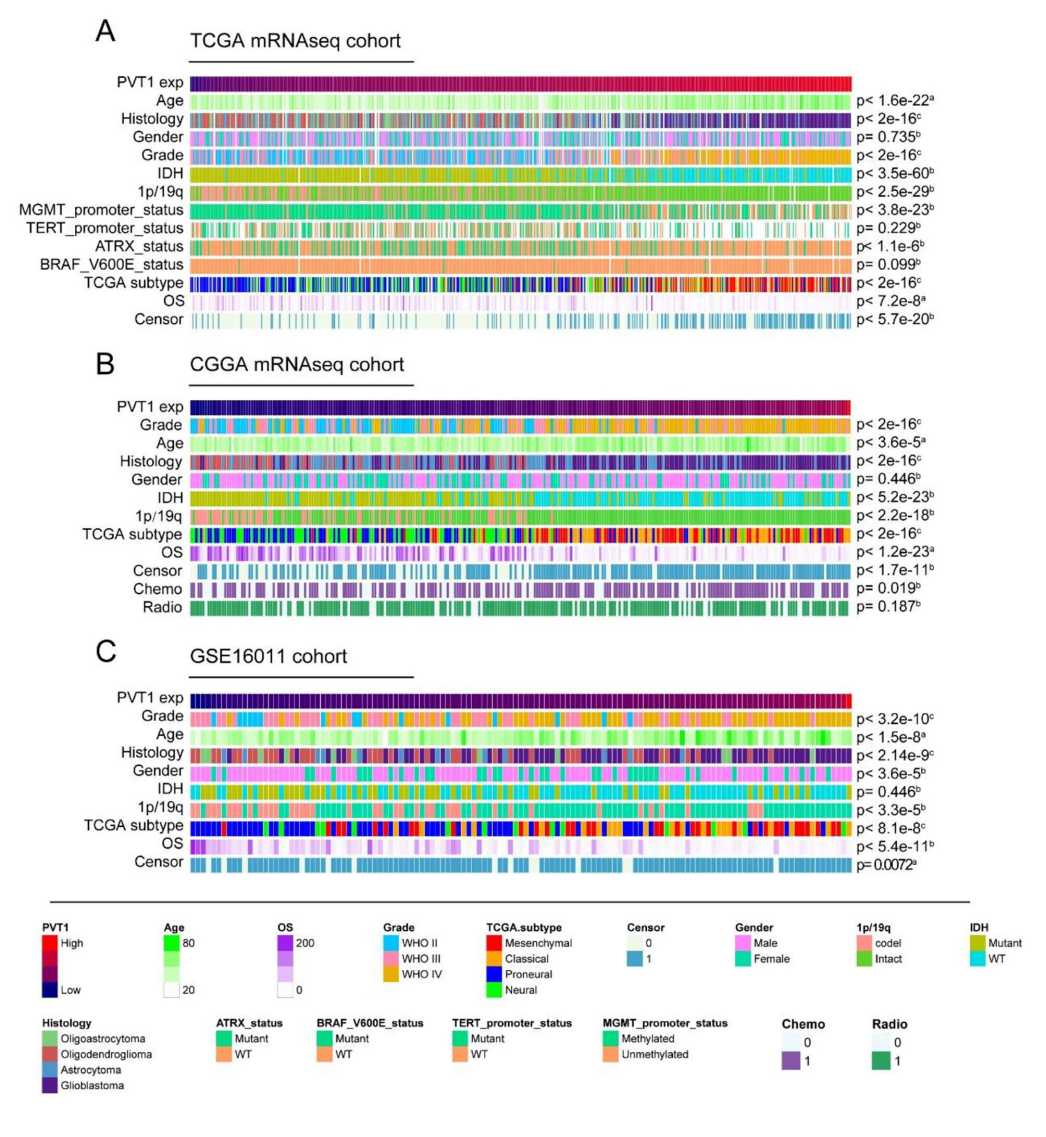
Landscape of clinical and molecular characteristics associated with PVT1 expression in gliomas. TCGA **(A)**, CGGA **(B)** and GSE16011 **(C)** cohorts were arranged in an increasing order of PVT1 expression. The relationship between PVT1 expression and patients’ characteristics was evaluated. (a, the association between PVT1 and continuous variables was assessed using Pearson correlation tests. b, the distribution of PVT1 was assessed using the Student’s t test between two groups. c, the distribution of glioma purity between several groups was assessed using one-way ANOVA)

To explore the relationship between PVT1 and clinicopathology characteristics, we arranged glioma samples according to the ascending order of PVT1 expression in three independent cohorts. As shown in Fig. 2, in three cohorts, we found that age at diagnosis was positively correlated with PVT1 expression (p < 0.0001), while a negative association was shown between PVT1 expression and overall survival (p < 0.0001). There was no difference of PVT1 expression between male and female. In addition, MGMT promoter (p < 0.0001) and ATRX status (p < 0.0001) rather than TERT promoter status (p = 0.23) and BRAF V600E status (p = 0.099) had a large impact on PVT1 expression in TCGA cohort. In CGGA cohort, PVT1 expression was significantly upregulated in patients under chemotherapy (p = 0.019), while not closely related to radiotherapy. It is well known that the MGMT promoter methylation status is the key biomarker to predict outcome in patients with temozolomide (TMZ) treatment [2, 14], and ATRX status correlate with alternative lengthening of telomeres (ALT) development [15]. Our result indicated that dysregulation of PVT1 expression was associated with genetic alterations.

### PVT1 predicted worse survival in gliomas

**Fig. 3:**
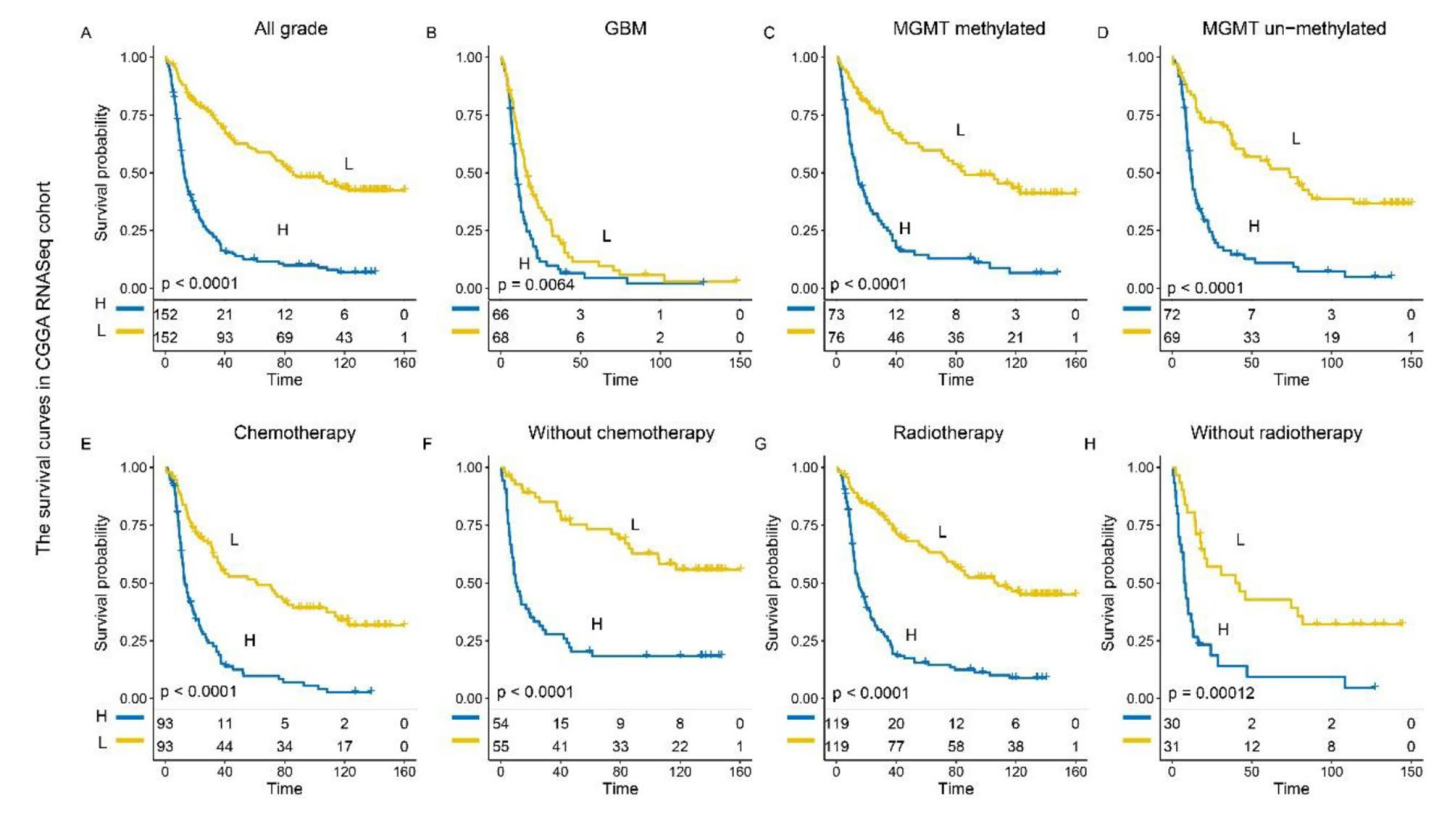
PVT1 was an unfavorable prognostic factor in glioma patients in CGGA cohort. **(A-B)** Kaplan–Meier survival analysis revealed a high level of PVT1 expression was an unfavorable prognostic factor in all grade patients and GBM patients. **(C-H)** Kaplan–Meier survival analysis based on PVT1 expression in stratified patients (MGMT methylated, MGMT un-methylated, chemotherapy, without chemotherapy, radiotherapy and without radiotherapy)

We further uncovered the prognostic significance of PVT1 across diffuse glioma in three cohorts. In CGGA cohort, the Kaplan-Meier analysis verified that gliomas with higher expression levels of PVT1 experienced significantly shorter overall survival (OS) in both whole gliomas (p < 0.0001) and GBM (p = 0.0064) (Fig. 3A, B). Meanwhile, the MGMT promoter methylated patients with higher PVT1 expression implied significantly inferior OS (p < 0.0001) (Fig. 3C), which suggested that PVT1 expression contributed to TMZ chemotherapy resistance. And MGMT promoter un-methylated patients with higher PVT1 expression had a shorter OS (p < 0.0001) (Fig. 3D). Then, patients were further stratified by treatment methods. Consequently, consensus results were found in patients with chemotherapy (p < 0.0001), without chemotherapy (p < 0.0001), radiotherapy (p < 0.0001) and without radiotherapy (p < 0.0001) (Fig. 3E-H). In addition, PVT1 expression was found as an unfavorable risk factor in TCGA and GSE cohorts (Fig S1). These results demonstrated that PVT1 expression was an unfavorable predictor for patients with glioma.

### PVT1 expression associates with distinct patterns of genomic alterations

**Fig. 4:**
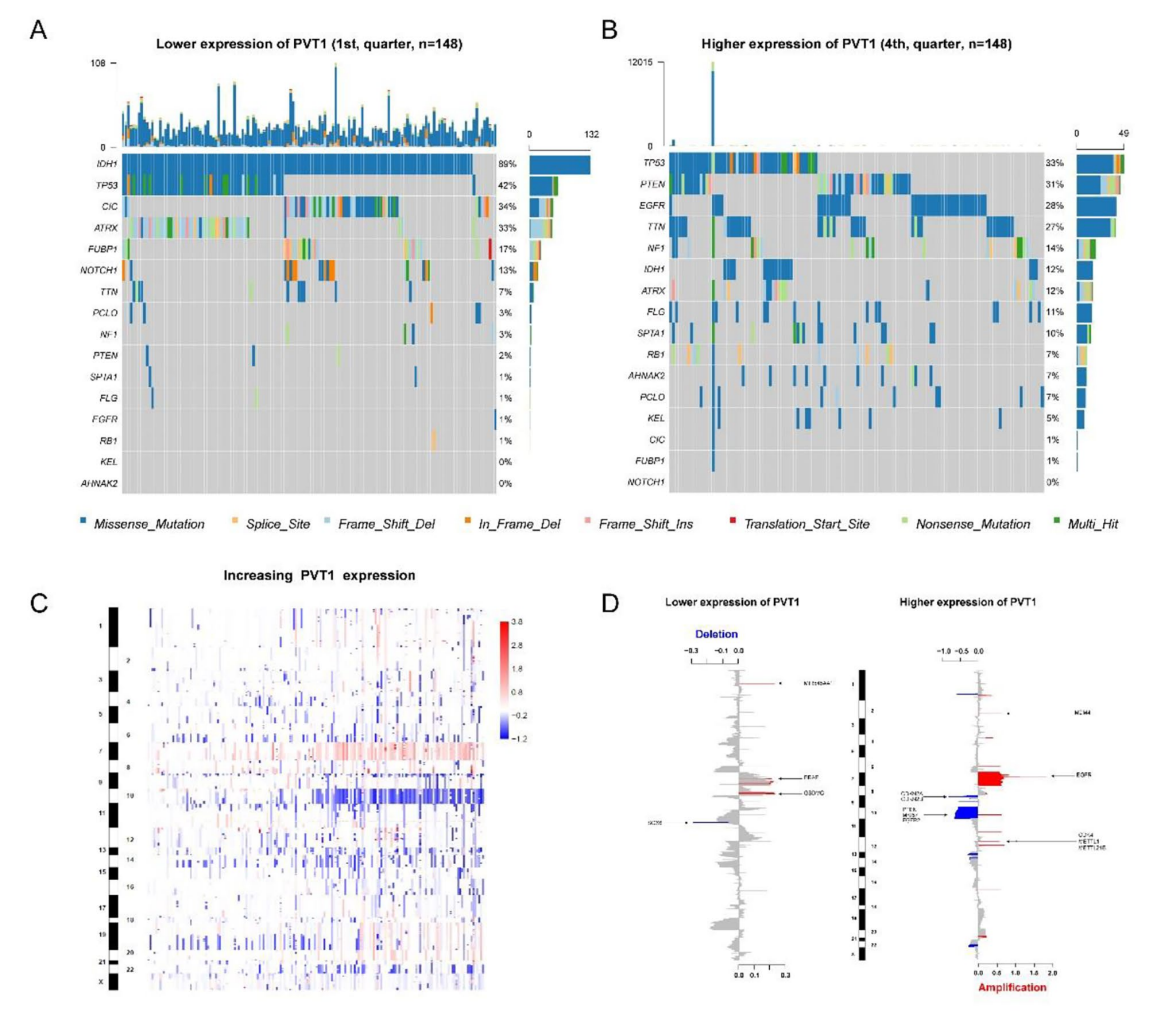
The genomic characteristics of PVT1 expression. **(A-B)** differential somatic mutations were detected by comparing gliomas with low and high PVT1 expression. **(C)** the overall CNVs profile in order of increasing PVT1 expression. **(D)** the CNVs alterations in low and high PVT1 expression groups. Chromosomal locations of peaks of significantly recurring focal amplification (red) and deletions (blue) were presented

To explore genomic alterations related to PVT1 expression, we analyzed the somatic mutation and copy number variation profiles from TCGA cohort. Based on the PVT1 expression, we stratified TCGA glioma samples by defining tumor in top 25th as higher PVT1 expression group and bottom 25th as lower group, and we found the mutation frequency of IDH1, TP53, CIC, ATRX, EGFR, PTEN, TTN, FUBP1, NOTCH1, SPTA1, KEL, FLG, NF1, RB1, PCLO and AHNAK2 were significant difference among the two group. Cases in PVT1 lower group were enriched in IDH1, CIC, FUBP1, NOTCH1 and TP53 mutation which were described in most common type of primary lower grade glioma (Fig. 4A). On the contrary, the mutation of PTEN, EGFR, TTN, NF1, SPTA1 and RB1, which occurred frequently in glioblastoma, were more frequently in PVT1 higher group (Fig. 4B).

Subsequently, CNV findings revealed distinct chromosomal alteration patterns between two groups. Chr 7 amplification accompanied with Chr 10 loss were enriched in the higher PVT1 expression cases (Fig. 4C). Meanwhile, in patients with higher PVT1 expression group, the most frequently deleted genomic regions were 9p21.3 encompassing CDKN2A/CDKN2B and 10q23.3 encompassing PTEN. And the most commonly amplified regions were 7p11.2 containing EGFR and 12q14.1 containing CDK4 (Fig. 4D). Taken together, PVT1 expression was associated with distinct features of genomic mutations.

### PVT1 related gene ontology in glioma

**Fig. 5:**
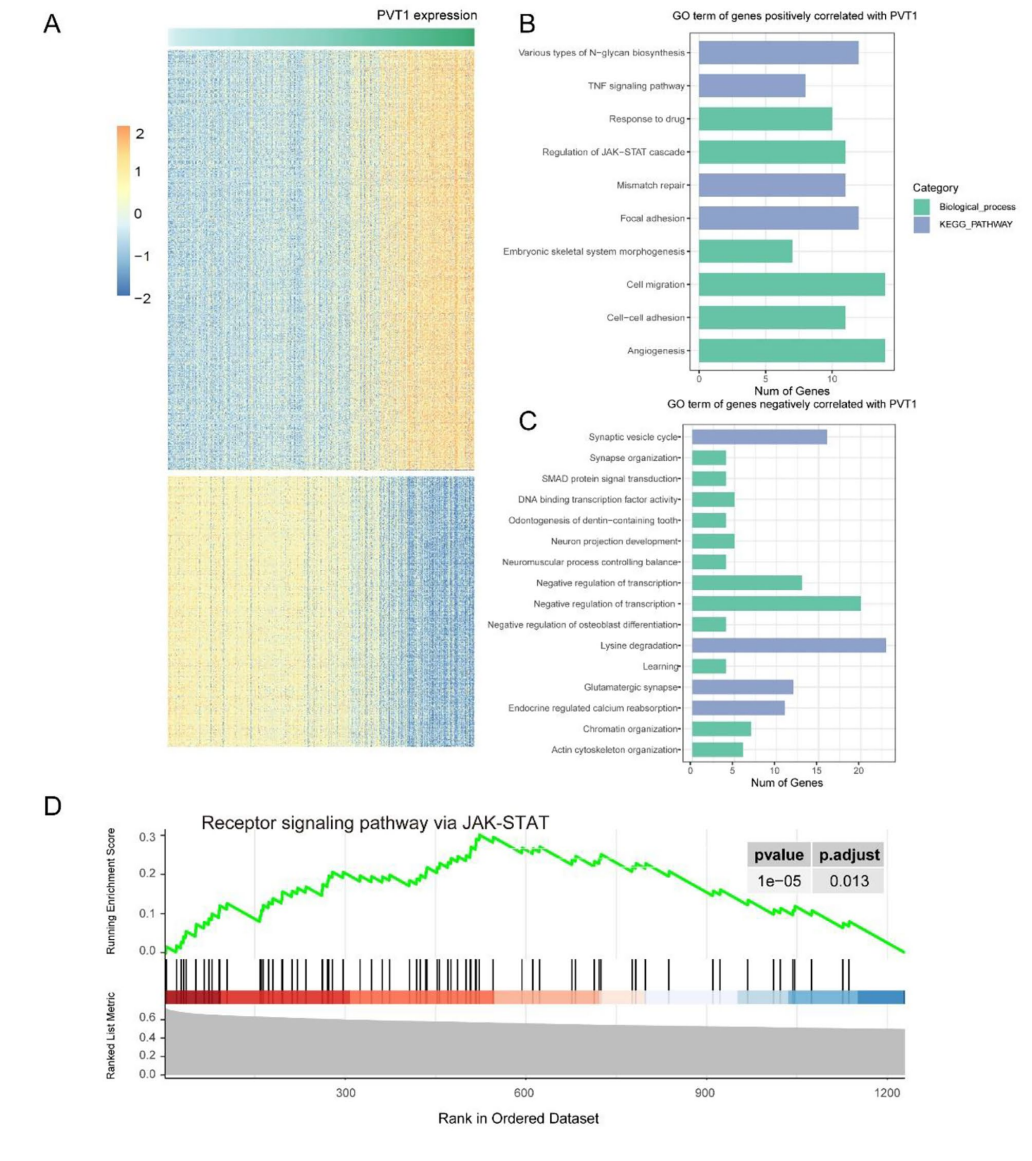
Gene ontology analysis of PVT1-associated genes in glioma. **(A)** Heatmaps showed genes positively (n = 408) and negatively (n = 215) corelated with PVT1 expression. **(B-C)**, GO and KEGG pathway analysis were performed to explore the functional annotations of PVT1 positively and negatively related genes. **(D)** GSEA method showed that JAK/STAT pathway was significantly enriched in patients with higher level of PVT1 expression (p.adj = 0.013)

In order to clarify the biological progress related to PVT1 expression, we obtained genes significantly correlated with PVT1 (Pearson |R| >0.5 and p < 0.05). PVT1 correlation genes, including 408 positively genes and 215 negatively genes, were shown in heatmap (Fig. 5A). We found that PVT1 positively correlated genes were mainly involved in various types of N-glycan biosynthesis, TNF signaling pathway, response to drug, regulation of JAK-STAT cascade, Mismatch repair (MMR), Focal adhesion, cell migration, cell-cell adhesion and angiogenesis (Fig. 5B). Additionally, the genes negatively correlated with PVT1 expression were mainly participated in basic functions of neuron, such as synapse organization and vesicle cycle, SMAD protein signal transduction, DNA binding transcription factor activity, odontogenesis of dentin-containing tooth, neuron projection development, neuromuscular process controlling balance and son on (Fig. 5C). The JAK/STAT pathway is the key pathway in transmitting chemical signals from extracellular to nucleus and participated in tumorigenesis and chemo-resistance [16]. The GSEA verified that PVT1 was closely associated with the function of JAK/STAT signaling (Fig. 5D). Taken together, the results implied that PVT1 might play a key role in glioma chemoresistance.

### Higher PVT1 expression was associated with TMZ resistance in glioma

**Fig. 6:**
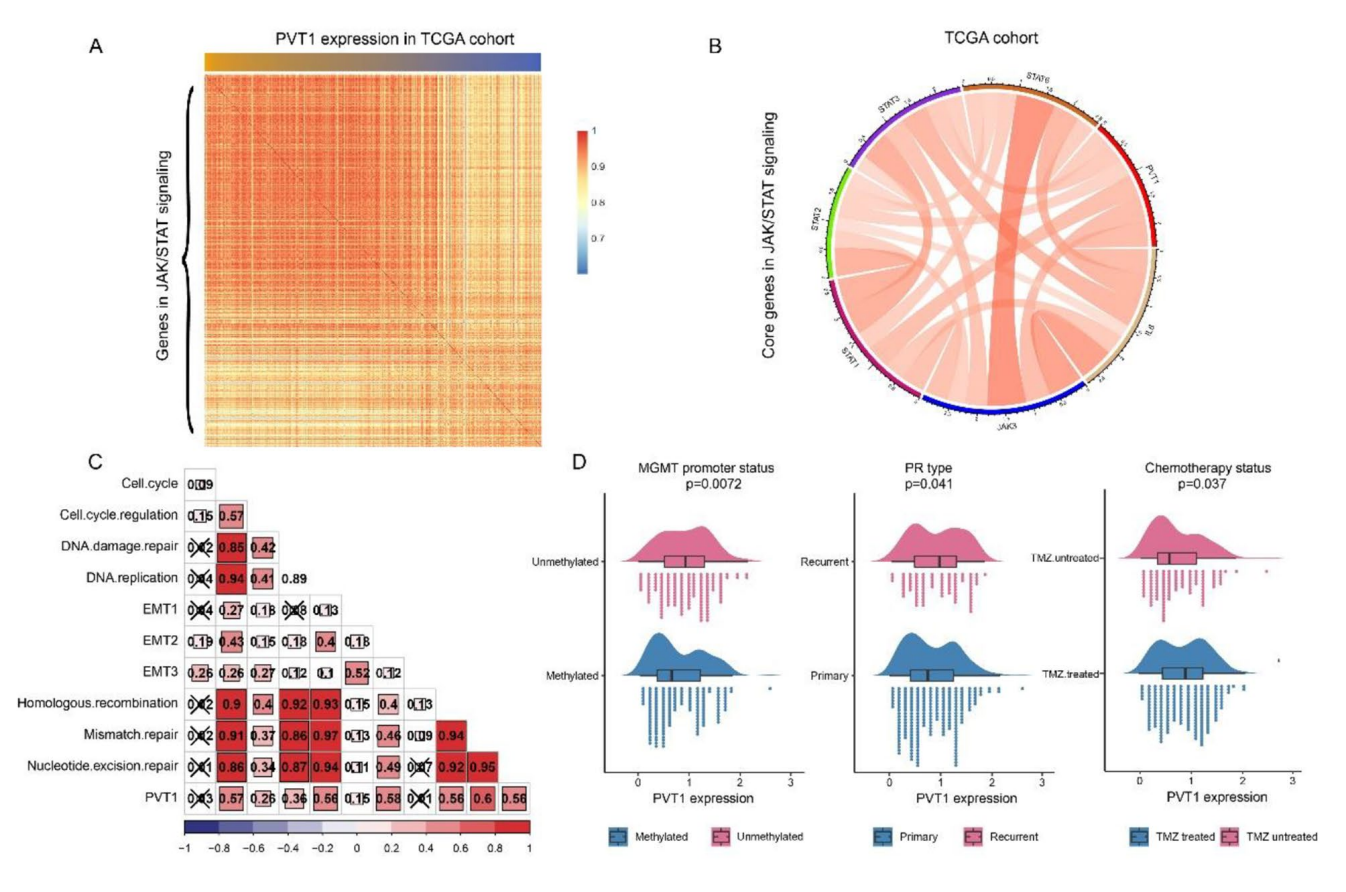
PVT1 was significantly correlated with JAK/STAT pathway and chemoresistance. **(A)** Heatmaps show the relationship between PVT1 and genes in JAK/STAT pathway. **(B)** PVT1 was positively correlated with IL6, STAT1, STAT2, STAT3 and JAK3. **(C)** The relationship between PVT1 expression and ten chemoresistance activities. **(D)** The relationships between PVT1 expression and MGMT promoter status, PR type and chemotherapy status, were evaluated

As depicted above, PVT1 participated in regulating JAK/STAT signaling in glioma. In order to find out potential regulation mechanism, we analyzed the relationship between PVT1 and JAK/STAT pathway by Pearson correlation method. As shown in Figs. 6 and 75 genes in JAK/STAT pathway were positively correlated with PVT1, while 12 genes were negatively correlated with PVT1 (Fig. 6A). Among those positively genes, the core genes in JAK/STAT pathway, including IL6, STAT1, STAT2, STAT3 and JAK3, were positively correlated with PVT1 expression (Fig. 6B). We hypothesized that PVT1 was a crucial regulator of JAK/STAT pathway in glioma, which in line with previous research in bronchial asthma [17].

Temozolomide (TMZ) is the first-line chemotherapy drug that has been used to treat newly diagnosed glioblastoma for over a decade. MMR pathway alteration in gliomas contributed to TMZ resistance [18]. Out of MMR pathway, DNA damage repair (DDR), DNA replication (DR), homologous recombination (HR) and nucleotide excision repair (NER) were important events involved in TMZ resistance [19, 20]. Our results showed that PVT1 expression was positively correlated with DDR, DR, HR and NER signalings. Meanwhile, PVT1 expression was tightly related with EMT and cell cycle regulation (Fig. 6C). Then we explored the PVT1 expression distribution according to MGMT promotor status, PR type and chemotherapy status in CGGA cohort. We found MGMT promotor unmethylated glioma (p = 0.0072), recurrent glioma (p = 0.041), or with TMZ chemotherapy (p = 0.037) patients had higher level of PVT1 expression (Fig. 6D).

### Knockdown of PVT1 inhibited glioma progression and increase sensitivity to TMZ

**Fig. 7:**
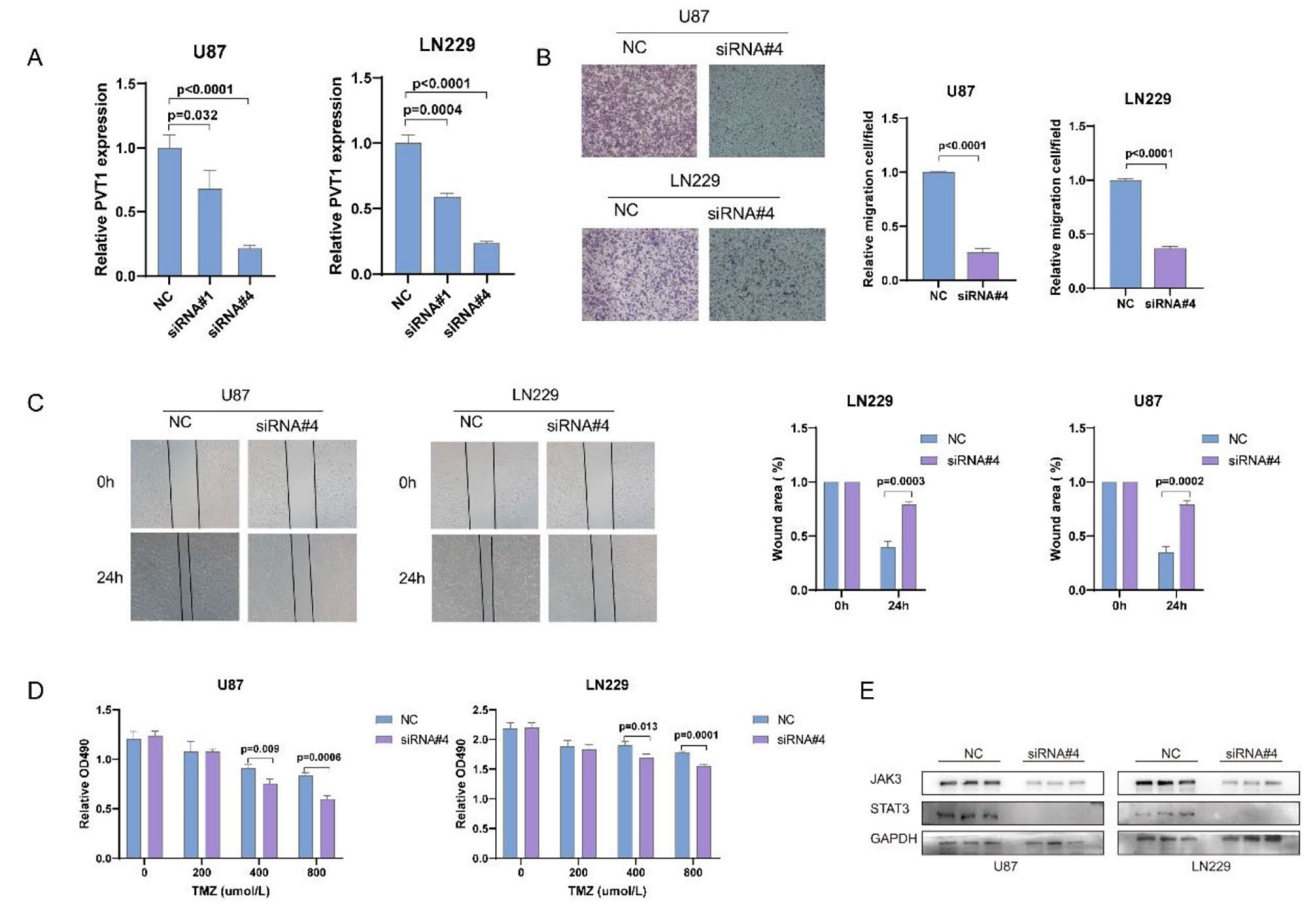
Knockdown of PVT1 inhibits cell proliferation and TMZ resistance in vitro. **(A)** qRT-PCR analysis of the knockdown efficiency of siRNA#1 and siRNA#4 in U87 and LN229; **(B)** Transwell assay of U87 and LN229 cell lines treated with PVT1 siRNA#4 or NC. **(C)** The wound-healing assays were performed to assess the effect of PVT1 knockdown on cell migration. **(D)** Knockdown of PVT1 increased the sensitivity of chemotherapy in TMZ-resistant U87 and LN229 cells. E, The protein level of JAK3 and STAT3 in U87 and LN229 cells were decreased after PVT1 knockdown

As the results indicated in functional and clinical analysis, PVT1 was involved in tumor progression and chemoresistance. We further experimentally verified the functional roles of PVT1 in glioma in vitro. The experiments of knockdown efficiency of siRNAs in U87 and LN229 indicated that siRNA-1 and siRNA-4 could significantly downregulated the expression of PVT1 (Fig. 7A). Transwell and cell scratch were performed in U87 and LN229 cell lines. We found that silence of PVT1 expression inhibited the migration ability of glioma cells (Fig. 7B ~ C). In additional, the CCK8 assay was used to assessed the cell viability. The assay results showed that the sensitivity of the cells to temozolomide (TMZ) was increased when PVT1 was knocked down, especially in the TMZ 400 and 800 µmol/L groups (Fig. 7D). Besides, the western blot analysis found that protein levels of JAK3 and STAT3 were dropped after PVT1 knockdown (Fig. 7E). These results suggested that PVT1 play a critical role in TMZ resistance and may mediate the activation of the JAK/STAT signaling.

## Discussion

Glioma is the most frequent malignant brain type of CNS in adults with poor prognosis [4]. The intratumor heterogeneity and the sensitivity to radiotherapy and chemotherapy contributed to the poor survival for glioma patients [21]. Therefore, understanding the molecular mechanisms underlying its progression behavior is urgently needed. Long non-coding RNAs are non-coding RNAs longer than 200 nucleotides. Numerous studies have shown that lncRNA play critical roles in translation and post-translational modification in glioma [22]. PVT1, at the 8q24 chromosomal band, were associated with cancer susceptibility and tumorigenesis [23]. However, there are few studies on the investigation the prognostic significance and clinical features of PVT1 in gliomas.

In this study, we collected the transcriptome and genomic data of 1210 glioma samples from CGGA, TCGA and GSE16011 cohorts to analyze the molecular and clinical characteristics of PVT1 comprehensively. Results showed that PVT1 expression was significantly increased with tumor grade in both three independent cohorts. Furthermore, PVT1 expression exhibited a tight relationship with classical and mesenchymal subtypes. These results were further validated by RT-PCR, which showed a high level of PVT1 expressed in the GBM tissue compared with the level in the LGG tissue. In addition, gliomas with chemotherapy tended to have a higher PVT1 expression. These findings showed that PVT1 contributed to the malignance of glioma.

To explore the mechanism of action of PVT1 in tumor progression in glioma, we investigated the distinct genomic alternations in order of increasing PVT1 expression.

We observed the events of somatic mutations and CNVs which were positively associated with PVT1 expression. Glioma with higher PVT1 expression was significantly associated with the somatic mutation of PTEN, EGFR and TTN. Meanwhile, the amplification of EGFR and CDK4, and the deletion of PTEN and CDKNA2A/B were observed in the gliomas with high PVT1 expression. The mutation rate of PTEN was 30 − 40% in GBM [24], and these findings have had a significant impact on management of PTEN mutant subtypes of glioma [25]. These findings suggested that PVT1 expression was associated with the malignant biological process. Exploring and revealing the mechanism of PVT1 in glioma may develop the therapeutic approaches to overcome this disease.

In addition, we found that patients with higher expression of PVT1 had a significantly shorter overall survival for glioma patients in each cohort. Our findings in line with the prognostic role of PVT1 in colon cancer [26, 27], gastric cancer [28] and melanoma cancer [29]. The results verified that PVT1 played an important role in the malignancy of glioma and may serve as a potential therapeutic target for glioma patients.

Pathway enrichment analysis showed that PVT1 was significantly related N-glycan biosynthesis, TNF signaling pathway, response to drug, regulation of JAK-STAT cascade, Mismatch repair (MMR). The JAK/STAT pathway is the key pathway in tumor progression and chemoresistance. The results were consistent with existing findings on the major physiological function of PVT1 in tumor development [10, 23, 30]. Then we depicted the relationship between PVT1, JAK/STAT pathway and chemoresistance. We found PVT1 expression was significantly positively related with genes in JAK/STAT pathway and DNA damage repair. Meanwhile, we found that PVT1 was highly expressed in recurrent GBMs, patients with chemotherapy, or patients with MGMT promoter unmethylated. As we know, the MGMT promotor was an important biomarker of tumor response to temozolomide (TMZ) chemotherapy [31]. The western blot analysis found that protein levels of JAK3 and STAT3 were decreased after PVT1 knockdown. Thus, we implied that PVT1 was not only functions as an important factor of the tumor proliferation but also results in the resistance of TMZ chemotherapy through regulating JAK/STAT pathway. The further mechanism and intervention treatment required extensive studies to validate in vivo. In the future, the combined strategy of TMZ and anti-PVT1 may improve the prognosis of GBM.

## Conclusion

Taken together, we elaborated the roles of PVT1 in glioma from transcriptomic and genomic levels. In addition, this study indicated that PVT1 promoted glioma TMZ chemoresistance through JAK/STAT signaling. The underlying mechanism provides a basis for PVT1 as a new molecular target for treatment of glioma.

## Electronic supplementary material

Below is the link to the electronic supplementary material.

Supplementary Material 1

## Data Availability

The datasets collected were the Chinese Glioma Genome Atlas (CGGA) database (http://www.cgga.org.cn), The Cancer Genome Atlas (TCGA) database (http://cancergenome.nih.gov/)[32].
