# Supplementary material for: LncRNA-PVT1 was identified as a key regulator for TMZ resistance and STAT-related pathway in glioma
Source: BMC Cancer. 2023 May 18;23:455. doi: 10.1186/s12885-023-10937-9 (PMC10197392; doi:10.1186/s12885-023-10937-9)

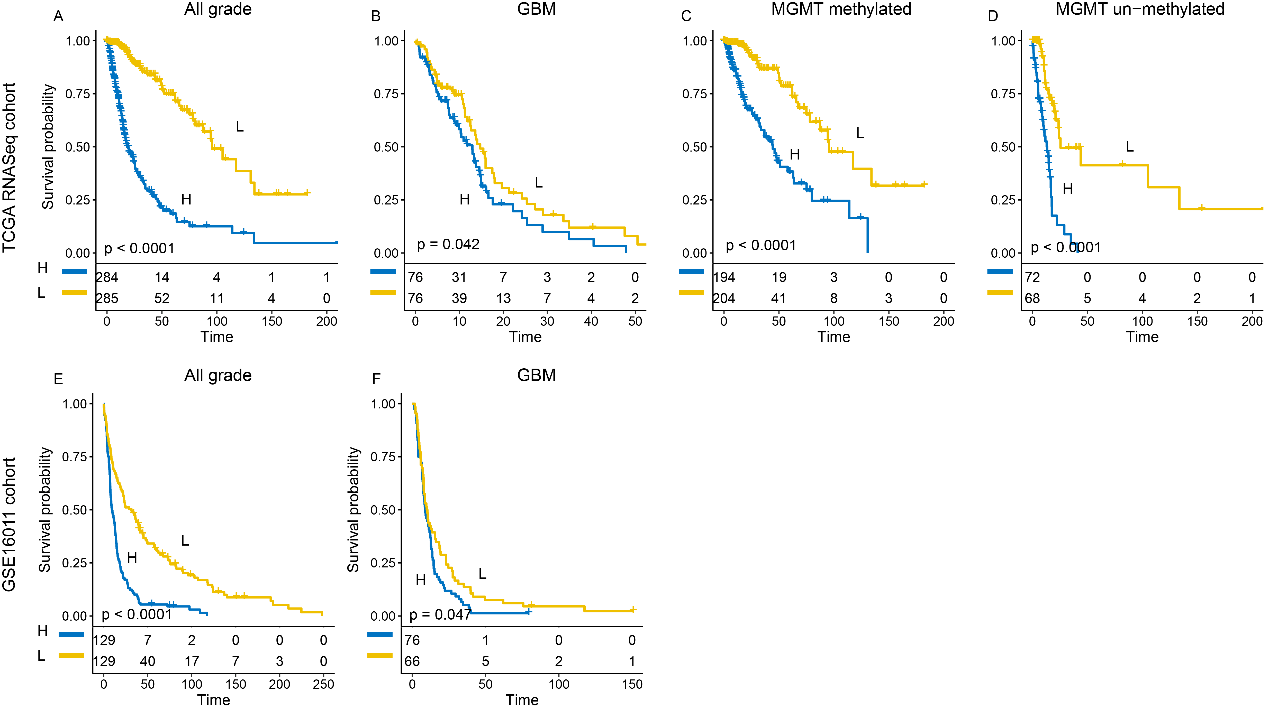


**Figure S1**

PVT1 was a prognostic factor in glioma patients TCGA and GSE16011 cohorts. (A) Kaplan–Meier survival analysis of all grades of glioma patients based on PVT1 expression in TCGA cohort. (B) Kaplan–Meier survival analysis of GBM patients based on PVT1 expression in TCGA cohort. (C) Kaplan–Meier survival analysis of patients with MGMT promoter methylation based on PVT1 expression in TCGA cohort. (D) Kaplan–Meier survival analysis of patients without MGMT promoter methylation based on PVT1 expression in TCGA cohort. (E) Kaplan–Meier survival analysis of all grades of glioma patients based on PVT1 expression in GSE16011 cohort. (F) Kaplan–Meier survival analysis of GBM patients based on PVT1 expression in GSE16011 cohort.

The original version of Western Blot.


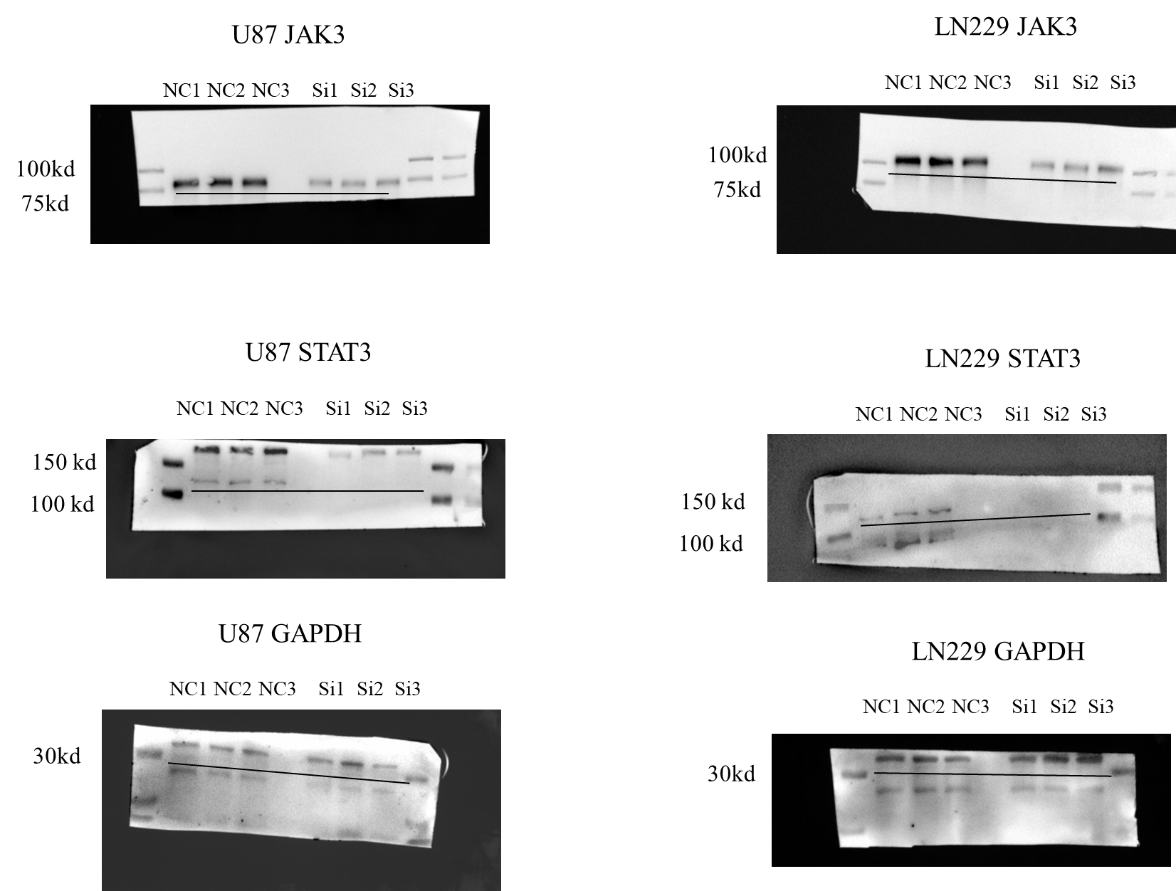

Supplement: Supplementary file 1 — Supplementary Material 1 [file 12885_2023_10937_MOESM1_ESM.docx]
